# Ionic contrast across a lipid membrane for Debye length extension: towards an ultimate bioelectronic transducer

**DOI:** 10.1038/s41467-021-24122-8

**Published:** 2021-06-18

**Authors:** Donggeun Lee, Woo Hyuk Jung, Suho Lee, Eui-Sang Yu, Taikjin Lee, Jae Hun Kim, Hyun Seok Song, Kwan Hyi Lee, Seok Lee, Sang-Kook Han, Myung Chul Choi, Dong June Ahn, Yong-Sang Ryu, Chulki Kim

**Affiliations:** 1 Sensor System Research Center, Korea Institute of Science and Technology, Seoul, Republic of Korea; 2 Department of Electrical & Electronic Engineering, Yonsei University, Seoul, Republic of Korea; 3 Department of Chemical and Biological Engineering, Korea University, Seoul, Republic of Korea; 4 Department of Bio and Brain Engineering, Korea Advanced Institute of Science and Technology (KAIST), Daejeon, Republic of Korea; 5 Center for Biomaterials, Korea Institute of Science and Technology, Seoul, Republic of Korea; 6 KU-KIST Graduate School of Converging Science and Technology, Korea University, Seoul, Republic of Korea

**Keywords:** Biosensors, Bionanoelectronics, Biosensors

## Abstract

Despite technological advances in biomolecule detections, evaluation of molecular interactions via potentiometric devices under ion-enriched solutions has remained a long-standing problem. To avoid severe performance degradation of bioelectronics by ionic screening effects, we cover probe surfaces of field effect transistors with a single film of the supported lipid bilayer, and realize respectable potentiometric signals from receptor–ligand bindings irrespective of ionic strength of bulky solutions by placing an ion-free water layer underneath the supported lipid bilayer. High-energy X-ray reflectometry together with the circuit analysis and molecular dynamics simulation discovered biochemical findings that effective electrical signals dominantly originated from the sub-nanoscale conformational change of lipids in the course of receptor–ligand bindings. Beyond thorough analysis on the underlying mechanism at the molecular level, the proposed supported lipid bilayer-field effect transistor platform ensures the world-record level of sensitivity in molecular detection with excellent reproducibility regardless of molecular charges and environmental ionic conditions.

## Abstract (web summary)

The design of bioelectronic devices that enables accurate detection of biomolecules in ionic solutions at physiologically-relevant concentrations remains a challenge. Here, the authors report a ion-impermeable supported lipid bilayer-assisted field-effect transistor platform for biomolecule detection.

## Introduction

**Fig. 1:**
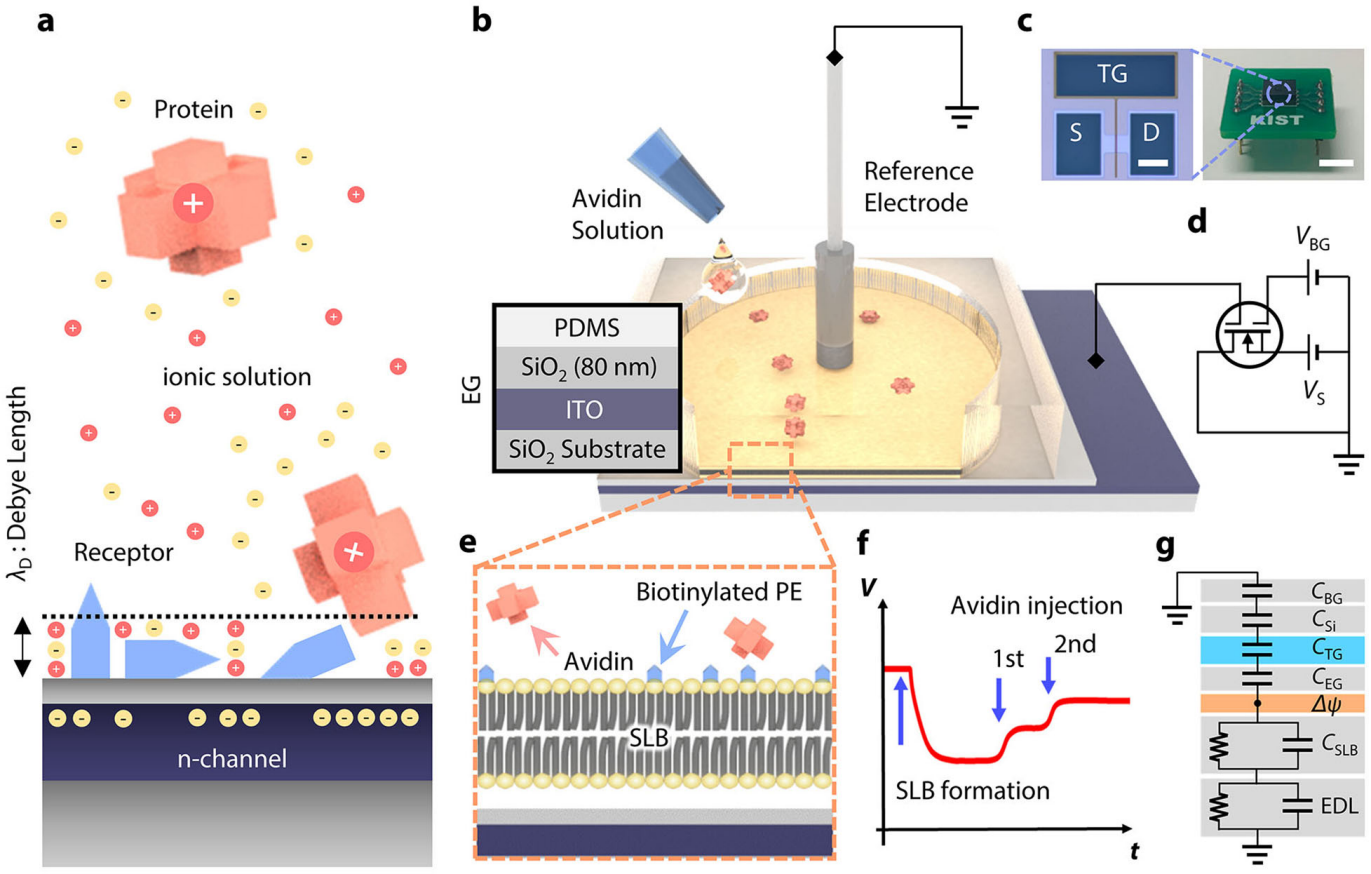
Electrical sensing in ionic environments and schematic of the SLB-assisted FET measurement setup. **a** Challenges to potentiometric measurement schemes for molecular detection on an FET under ionic environment: formation of the EDL, non-specific binding, and randomly oriented receptors. **b** Schematic representation of a reaction chamber on the EG. (left) Layer-by-layer configuration of the EG. Polydimethylsiloxane (PDMS) chamber acts as a reaction chamber. **c** Optical images of the FET (left) and a packaged device (right) (scale bars = 100 μm and 5 mm, respectively). **d** Schematic representation of measurement setup. **e** Cross-sectional illustration of the SLB on the EG surface. **f** Proof-of-concept for real-time detection during biotin–avidin binding. **g** Equivalent circuit model for the measurement setup.

Detection of molecular interactions between biomolecules and their counterparts, including antibody–antigen complexes^1,2^, complementary single-stranded deoxyribonucleic acid^3,4^, and enzyme–substrate interactions, has been demonstrated by various transducers with target-specific functionalization^5^. Detection of molecules via surface-sensitive bioelectronics has allowed for portable biomedical devices to implement point-of-care diagnostics to prescreen diseases without temporal and spatial limitations^6^. Among them, field-effect transistors (FETs) has attracted considerable attention because of their strength in miniaturization, cost-effective mass production, and seamless integration with manufacturing processes. Potential variations caused by molecular adsorption/desorption on the probing surface are effectively transduced into carrier density variation in the active current channel of FETs^7^, allowing “real-time” molecular detection without cumbersome labeling or signal-amplifying processes^8^. Despite the significant potential, molecular detection with FETs in ionic solutions at physiologic concentrations remains an ongoing challenge, mainly because of the formation of an “electrical double layer (EDL)” over the active sensing probe^9^ (Fig. 1a). Formation of the EDL originates from the fact that analytes electrically attract counter ions from an ionic solvent, causing exponential decay of electrical potential with distance. This screening effect inevitably limits the detection of induced potential variation arising from molecular interaction. For example, the effective detection range, termed Debye length (*λ*_D_), becomes <1 nm in physiological conditions^10^. In addition, the non-specific binding of target molecules to the probe degrades sensor reliability by disrupting their selective sensing principles. Thus, a novel strategy that can overcome the aforementioned hurdles is in high demand for unraveling the physicochemical nature of molecular interaction with biosensors.

Diverse efforts have been undertaken by facilitating advanced measurement scheme and functional materials such as heterodyne detection^11^, antibody fragments^12^, and deformable aptamers^13^, just to mention a few. Although these approaches shed light on molecular detection in ionic solutions, they still have certain limitations or require enhancements. The heterodyne detection, which results in complexity of the operating circuitry, was not functioned under physiological ionic strength conditions. Antibody fragments are placed on the probe surface with random orientations, which often degrades measurement reproducibility. Although deformable aptamers resolve previous shortcomings, aptamers require intensive work for their specific design. Along this line of thought, an ideal platform for biosensing applications is considered to, especially in physiological ionic strength conditions, be far from being established.

Here, we propose an ion-impermeable SLB-assisted FET (SLB-FET) platform for molecular detection with a high degree of consistency irrespective of environmental ionic conditions. The potential of supported lipid bilayers (SLBs), a mimicry of cellular membranes, as a bioelectronic transducer has been glimpsed^14^. Such a prospective conjecture in their biosensing applications originates from their nature: (i) high resistivity to non-specific protein adsorption^15^, (ii) surface passivation with ion impermeability^14^, and (iii) morphological preservation of receptors^16^. By turning the fascinating glimpse into the realization, we demonstrate robust molecular detection under biologically relevant conditions, characterize its sensing capability in terms of sensitivity and selectivity, and finally investigate the signal transfer mechanism in our SLB-FET platform, which is triggered by real-time molecular bindings.

## Result

### Fabrication and measurement setup

The measurement setup for molecular detection comprises an FET and a reaction chamber placed on an extended gate (EG) (Fig. 1b, d). We fabricated FETs with an active current channel in nanoscale by facilitating conventional semiconductor fabrication techniques (Fig. 1c and Supplementary Fig. 1). The thickness ratio of the top (14 nm) and bottom (750 nm) oxide layers determines its figure of merit^17^. The top gate electrode is electrically connected to an indium tin oxide (ITO) layer working as the EG electrode (see Supplementary Note 1 for details), wherein the gate potential change modulates the conductance in the FET. This allows for reliable data acquisition by eliminating any possible contamination or damage to the active channel of the FET. Furthermore, all the responses are transduced by an identical FET device, which enables direct comparison of individual signals measured with different analytes at various concentrations. To maximize the response amplitude, the back-gate voltage (*V*_BG_) was adjusted to ensure the maximum slope in the top gate response curve (Supplementary Fig. 3).

### Real-time monitoring of SLB coverage

**Fig. 2:**
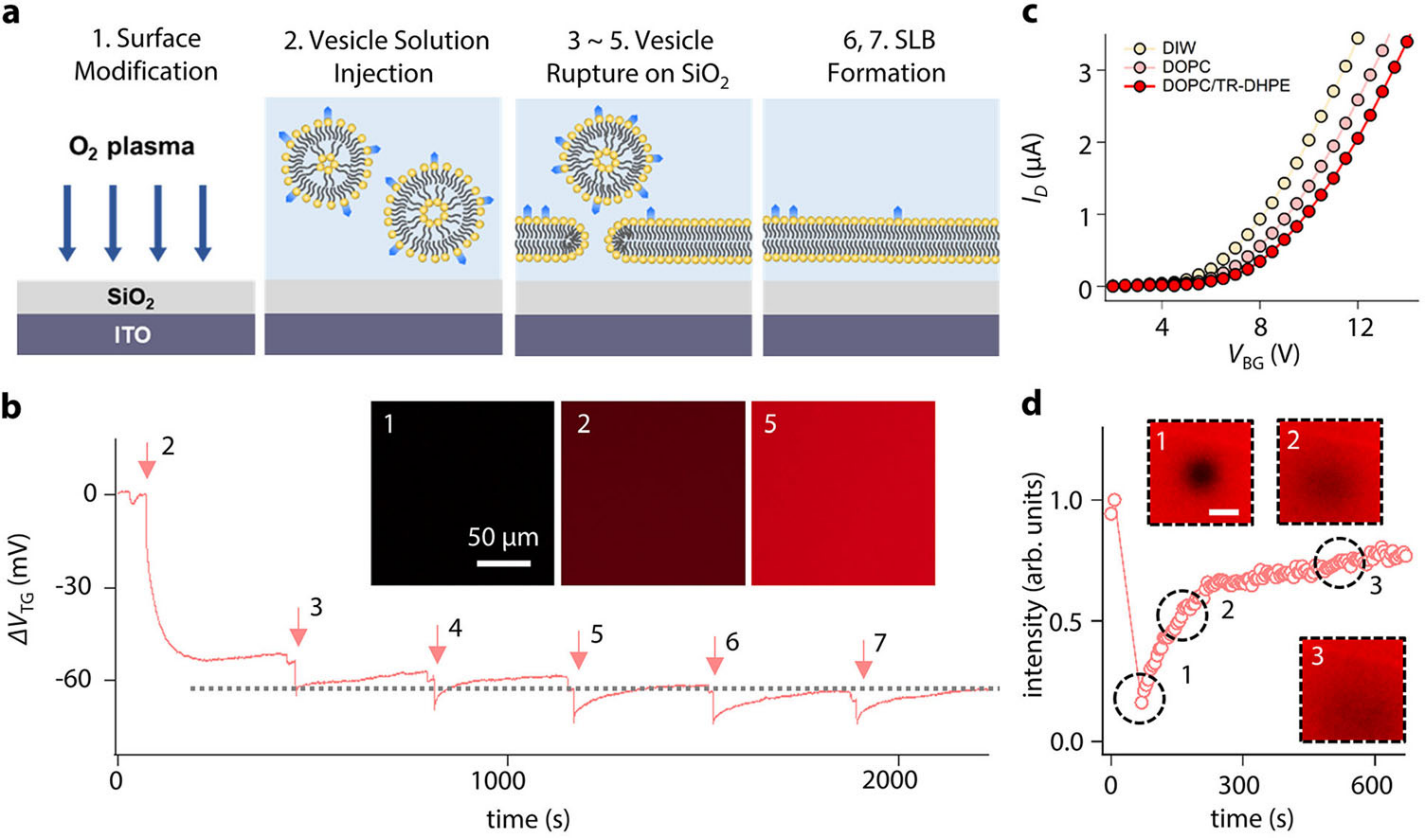
SLB formation and its time-dependent measurement. **a** Schematic illustration of SLB formation. **b** Real-time tracing during an SUV rupture process. Insets show fluorescent images of the EG after SLB formation at different vesicle concentrations; (marker 1) after O_2_ plasma treatment, (marker 2) at 1 μg mL^−1^, and (marker 6) at 5 μg mL^−1^. **c** *I*_D_–*V*_BG_ curves with different ingredients in the SLB. **d** Time-dependent FRAP measurement. The scale bar in the inset is 50 μm. Source data are provided as a Source Data file.

SLB formation by spontaneous rupture of small unilamellar vesicles [SUV; 1 μg mL^−1^ in deionized water (DIW)] was examined using the FET (Fig. 2a). The SUV solution comprises 95% 1,2-dioleoyl-*sn*-glycero-3-phosphocholine (DOPC) and 5% 1,2-dioleoyl-*sn*-glycero-3-phosphoethanolamine-*N*-(cap biotinyl) (B-PE). Figure 2b shows the potential variation of the top gate electrode converted from the obtained current during the SLB formation (see “Methods” section for details). The observed saturation after the fourth injection of the SUV is consistent with the calculation that 0.4 μg of lipids is sufficient to completely cover the SiO_2_ surface of the EG (see Supplementary Note 5 for details). An epifluorescence microscopy was used for the cross-validation of the SLB formation (the inset of Fig. 2b).

Figure 2c shows drain current (*I*_D_) as a function of *V*_BG_ with different SLB compositions, for direct comparison with compositional variations. The graph displays a signal resulting from the SLB containing 1 mol% of negatively charged Texas Red 1,2-dihexadecanoyl-*sn*-glycero-3-phosphoethanolamine (TR-DHPE)^18^ and shows a positive threshold voltage shift compared to the neutral SLB (DOPC 100%). Note that our SLB-FET platform demonstrates sufficient molecular sensitivity to distinguish compositional differences within the SLB. For a fluorescence recovery after the photobleaching (FRAP) test, we prepared a sample with the mol% ratio of 94:5:1 of DOPC:B-PE:TR-DHPE. The bleached region was recovered to 80% level of the initial fluorescent intensity after 10 min (Fig. 2d). With the lateral diffusion property of the lipids and the SLB with this compositional ratio, two effective binding sites of single avidin are assumed to be occupied avoiding steric hindrance^19^. The diffusion rate was calculated to be 1.43 ± 0.4 μm^2^ s^−1^ with high uniformity over the SiO_2_-coated EG^20,21^.

### Measurements of biotin–avidin bindings and analytical models

**Fig. 3:**
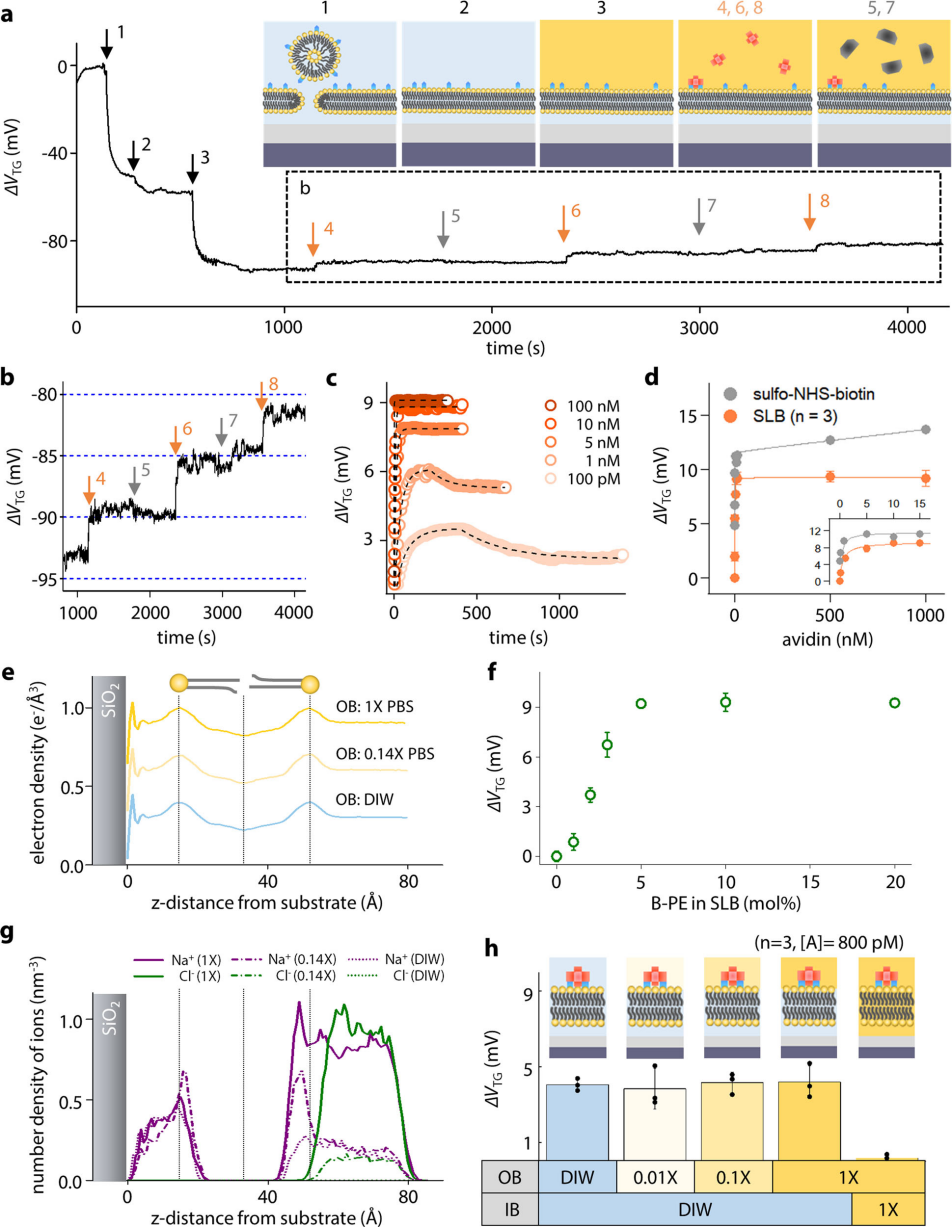
Measurements of biotin–avidin bindings using SLB-FET. **a** Real-time measurement for SLB formation and sequential molecular bindings. The inset illustrates different measurement stages. **b** Enlarged graph in **a** showing binding selectivity to avidin. **c** Real-time traces (circle) from biotin–avidin binding at different concentrations. Dashed lines represent fits by an analytical model. **d** Response amplitudes as a function of avidin concentration in the presence (orange)/absence (gray) of the SLB. The reported values of the SLB experiment represent the mean ± standard deviation (SD) for *n* = 3 independent experiments. **e** Electron density profile of supported lipid bilayer with different OB ionic concentrations obtained from the MD simulation. **f** Δ*V*_TG_ at different concentrations of B-PE expression in the SLB with the constant number of avidin binders. The reported values of the SLB experiment represent the mean ± standard deviation (SD) for *n* = 3 independent experiments. **g** MD simulation results of ion distribution of (Na^+^ and Cl^−^) at different concentrations. Note that significant Na^+^ peaks at *z* = 10 and 50 Å results from the lipid head group of the B-PE. **h** Sensor responses to 800 pM-avidin under different ionic conditions around the SLB. The measurement was performed for *n* = 3 independent experiments and all reported values represent the mean ± SD. Source data are provided as a Source Data file.

To evaluate our SLB-FET as a biosensor operating in an ionic solution, we conducted time-lapse measurements during biotin–avidin binding. The SLB containing 5% of B-PE with 95% of DOPC was prepared on the EG surface^19^. After SLB deposition through SUV rupture in DIW, the outer buffer (OB) solution above the SLB was exchanged with 1× phosphate buffer saline (PBS) to mimic physiologic conditions. Note that the ion impermeability of the SLB preserves the ionic imbalance across the lipid membrane (Supplementary Figs. 4 and 5). Figure 3a shows a typical real-time trace obtained from sequential steps including SLB formation (markers 1 and 2), OB exchange from DIW into 1× PBS (marker 3), 800 pM-avidin injections (markers 4, 6, and 8), and 800 pM-Cholera toxin subunit B (CTxB) injections (markers 5 and 7). CTxB was applied as a negative control analyte. While CTxB injections showed no response, avidin injections led to clear changes of the top gate voltage (Δ*V*_TG_ ~ 4.7 mV in the first injection) (Fig. 3b).

The real-time responses of biotin–avidin bindings at different analyte concentrations ranging from 100 pM to 100 nM are shown in Fig. 3c and summarized in Fig. 3d. To account for the obtained responses, an analytical model was suggested by combining the Langmuir isotherm model with the information of capacitively induced charges on the gate electrode of the FET. The induced voltage change Δ*V*_TG_ can be described as

1 $$\triangle {V}_{{\rm{TG}}}\left([A]\right)=\frac{{q}_{A}}{{C}_{{\rm{TG}}}}{\left[B\right]}_{{\rm{max }}}\frac{[A]}{\left[A\right]+{K}_{{{\mathrm{eq}}}}}$$

where *q*_*A*_ is the effective charge induced by molecular adsorption, [*B*]_max_ is the number of binding sites on the lipid membrane, [*A*] represents the analyte concentration, and *K*_eq_ is the equilibrium constant^22^. The *K*_eq_ of 621.9 pM for biotin–avidin binding was determined from the fitting using Eq. (1) (solid line in orange, Fig. 3d), which is consistent with the literature^19,23^. To determine binding parameters, a resistor-capacitor (RC) circuit model was combined with the first order Langmuir adsorption equation (see Supplementary Note 7 for details). The time-dependent Δ*V*_TG_ is then expressed as

2 $$\triangle {V}_{{\rm{TG}}}\left(t\right)=\left\{\begin{array}{cc}\frac{{q}_{{{A}}}}{{C}_{{\rm{TG}}}}{\left[B\right]}_{{\rm{max }}}\left(1-{e}^{-\left({k}_{1}\left[A\right]+{k}_{-1}\right)t}\right)+{V}_{{{p}}}(1-{e}^{-\frac{t}{{\tau }_{1}}}) & t < T\\ \frac{{q}_{{{A}}}}{{C}_{{\rm{TG}}}}{\left[B\right]}_{{\rm{max }}}\left(1-{e}^{-\left({k}_{1}\left[A\right]+{k}_{-1}\right)t}\right)+{V}_{{{p}}}{e}^{-\frac{t}{{\tau }_{2}}}\hfill & t\ge T\end{array}\right.$$

where *k*_1_ and *k*_−1_ are the association and dissociation rate constants, respectively, *V*_p_ is the difference between the maximum sensor response (*q*_*A*_[*B*]_max_/*C*_TG_) and the maximum value of Δ*V*_TG_(*t*), and *τ*_1_ and *τ*_2_ are the RC time constants. The rate constants were calculated to be *k*_1_ = 1.64 ± 0.06 × 10^7^ M^−^^1^ s^−1^ and *k*_−1_ = 1.02 × 10^−2^ s^−1^ based on fitting with the obtained value of *K*_eq_ (=*k*_−1_/*k*_1_) above (Fig. 3c). The response curve from the EG functionalized by biotin at an identical concentration without the SLB (circle in gray, Fig. 3d) shows a linear increase in the high concentration regime, which is attributable to the non-specific binding^24^. In contrast, this non-specific binding behavior was strongly suppressed by the SLB (Fig. 3d orange, and Fig. 3f). We conducted molecular dynamics (MD) simulation to investigate the possibility of conformational change in the SLB under different OB conditions (Fig. 3e, g). From Fig. 3e, we concluded that the SLB preserved its structure under different ionic strengths. In addition, titrations of avidin at a fixed concentration (10 nM) to the gradually increased B-PE concentration (0, 1, 2, 3, 5, 10, and 20 mol%) were tested out in identical buffer conditions (IB for DIW, and OB for 1× PBS). A drastic increase of the Δ*V*_TG_ with respect to the B-PE concentrations (0 → 5 mol%) was observed. The saturated amplitude of Δ*V*_TG_ ≈ 9.3 mV at higher concentrations was well agreed with the results shown in Fig. 3d. From those, we confirmed that the SLB plays essential roles not only as a “protector” against charge-assisted binders including non-specific bindings, but also as an “ion keeper” enabling reliable signal acquisition under ionic outer buffer solution.

Figure 3h presents the sensor responses using different conditions for the buffer solution (OB: DIW, 0.01×, 0.1×, and 1× PBS, IB: DIW, and 1× PBS) around the SLB. Unlike the usual presumption that the sensing capability highly depends on the ionic strength of the solution, the responses to 800 pM avidin in different buffer conditions were measured alike within the error range. The control experiment with 1× PBS buffer solution underneath the SLB inevitably demonstrates the importance of the ionic condition of the inner buffer (IB) in our SLB-FET sensing mechanism. In addition, [*B*]_max_ and *C*_TG_ are calculated to be 1.74 × 10^8^ and 0.37 pF respectively in 5 mol% of the B-PE. With this, *q*_*A*_ is obtained to be 2.59 × 10^−23^ C (≈1.61 × 10^−4^ electrons) from the fitting. This suggests a significant discrepancy with the previous result of about 3.43 electrons for a single avidin molecule in 1× PBS solution^25^. These counter-intuitive results exhibiting ionic strength-independent FET signals led us to the following hypothesis: there could be a possible conformational change upon molecular binding, redistributing the electron density within the SLB.

### Analysis on the electron density change in the SLB upon molecular bindings

**Fig. 4:**
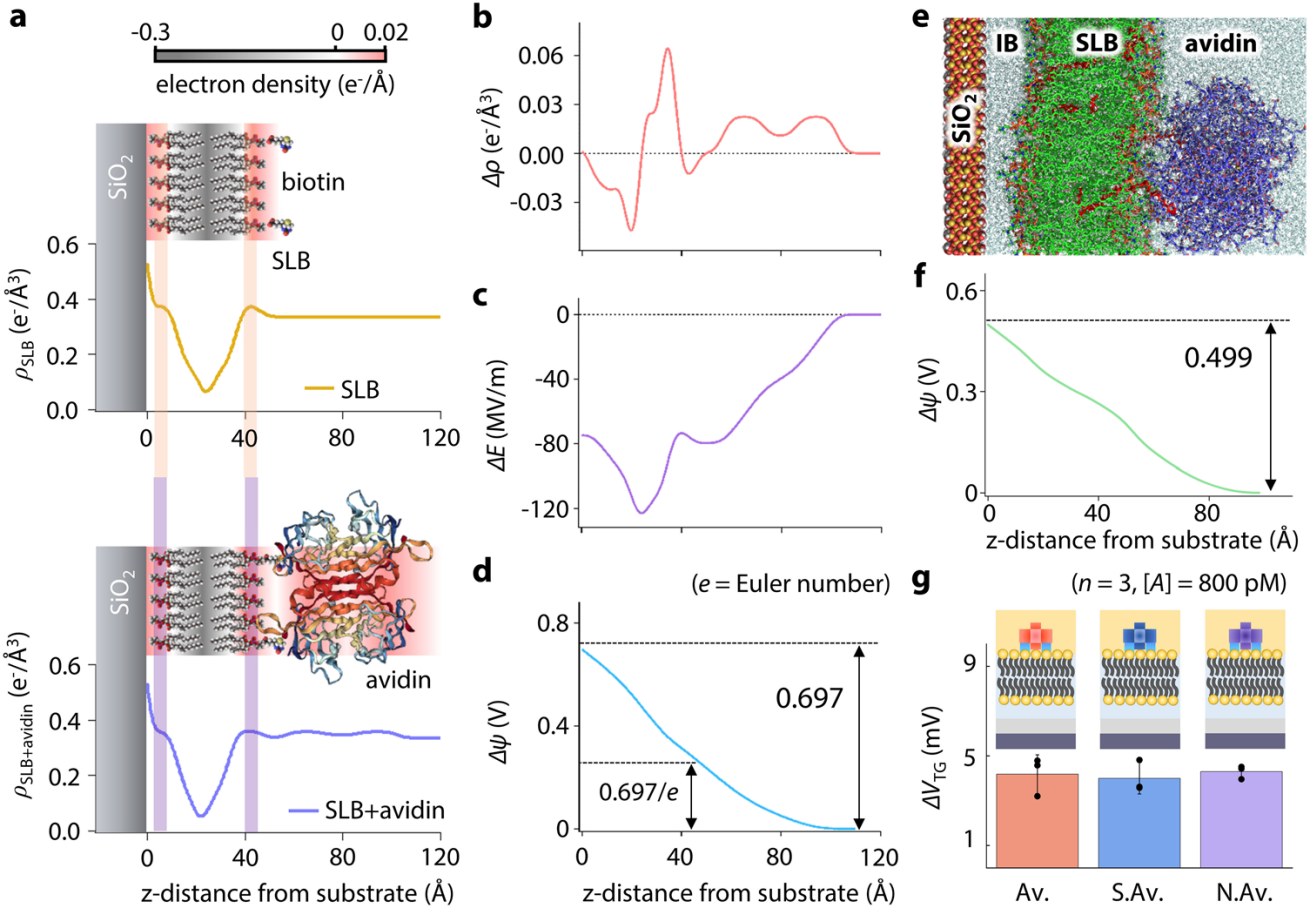
Redistribution of the electron density in the SLB upon biotin–avidin bindings. **a** Electron density profiles before and after biotin–avidin bindings. **b** Electron density difference (Δ*ρ* = *ρ*_SLB + avidin_ − *ρ*_SLB_). **c** Electric field change. **d** Chemical potential difference upon biotin–avidin binding. **e** MD simulation of the SLB under avidin binding. The DOPC lipids, B-PE lipids, avidin, and water molecules are colored in green, red, purple, and cyan, respectively. **f** Induced potential difference upon biotin–avidin binding obtained from MD simulation (see Supplementary Note 11 for details). **g** Sensor responses to different analytes at identical concentrations. (Av. avidin, S.Av. streptavidin, and N.Av. neutrAvidin). The measurement values represent the mean ± SD for *n* = 3 independent experiments. Source data are provided as a Source Data file.

To investigate the underlying sensing mechanism of our SLB-FET upon molecular binding, synchrotron X-ray reflectometry (XRR) was applied to acquire electron density profiles across the SLB (DOPC/B-PE = 95/5 in DIW) before/after avidin bindings^26^. The obtained electron density profiles from the reflectivity fits are presented in Fig. 4a (see Supplementary Fig. 9b for details). Despite the structural similarity, conformational changes to the free-standing SLB due to avidin binding are distinguishable, and are accompanied by the redistribution of electrons within the SLB. For quantitative analysis, differences in the electric field (∆*E*) and chemical potential (∆*ψ*) were calculated using Poisson’s equation (see Supplementary Note 9 for details) (Fig. 4b, c, and d). The induced chemical potential difference, ∆*ψ*, via the avidin binding was 697 mV from XRR and 499 mV from MD simulation at the surface of the SiO_2_ layer (Fig. 4d, f). Considering a series of potential drops along the circuit (Fig. 1g), the potential change at the top gate was calculated to be 12.2 and 8.73 mV respectively. This is consistent with the experimentally measured value, *q*_*A*_[*B*]_max_/*C*_TG_ of 9.38 ± 3.08 mV in the FET, considering the fact that the XRR experiment was conducted under DIW conditions (EDL_DIW_ » EDL_1× PBS_). Note that the ∆*V*_TG_ caused by the charges above the SLB (>55 Å in Fig. 4b) is within the range of standard deviation in the FET measurements. To further cement our understanding, we compared the signal responses of avidin variants (neutrAvidin: pI. 6.3, streptavidin: pI. 5–6, and avidin: pI. 10.5) with different isoelectric points (pIs) under 1× PBS at the identical concentration of 800 pM (Fig. 4g). Coterminous FET responses regardless of different pIs support our finding that the majority of effective signals originate from dipole field change within the SLB (see Supplementary Note 11 for details), not from the conjugated proteins themselves^27^.

## Discussion

**Fig. 5:**
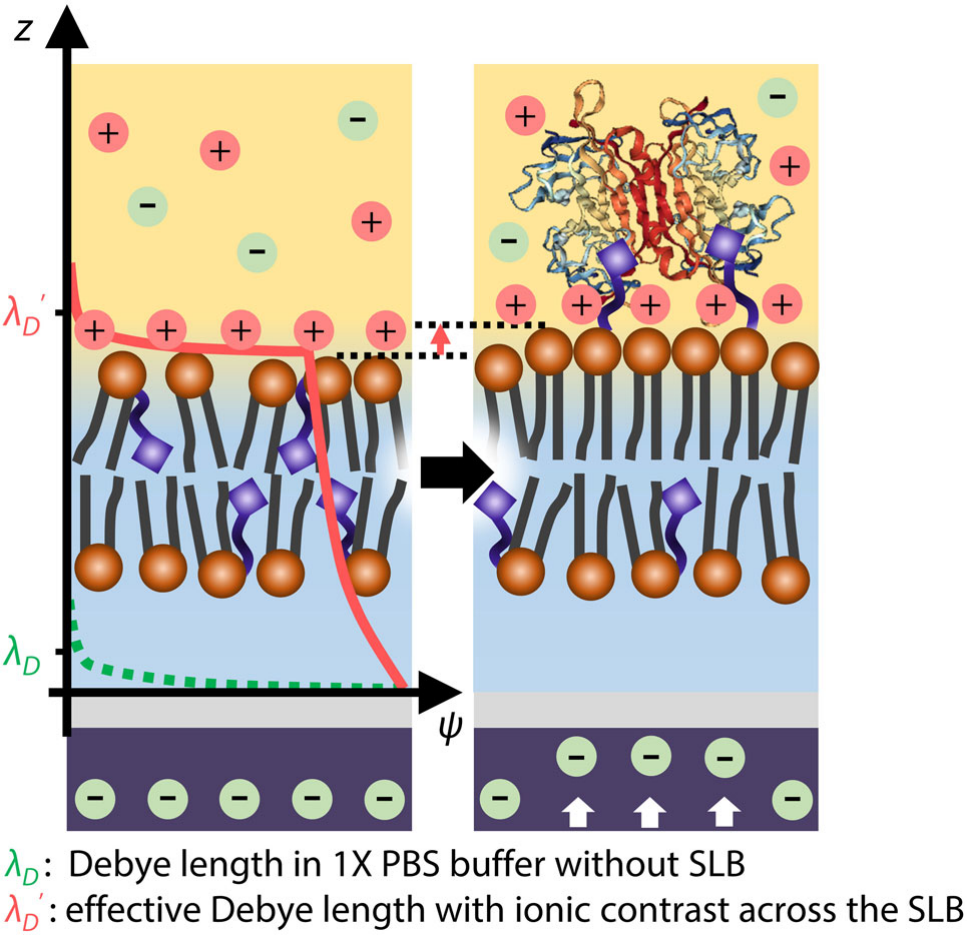
Improved molecular detection via asymmetric ionic environment across the SLB. Schematic illustration of effective Debye length (*λ*_D_′) with ionic contrast across the SLB and conformational change upon avidin binding.

Most hitherto electrical sensing of molecules required buffer dilution to weaken the ionic strength of solutions. However, the structural integrity and molecular activity of target analytes are not guaranteed in such attenuated ionic environments. Previous experiments with the SLB over probing materials showed an inevitable loss of sensitivity due to the screening effect by ion accumulation between the probing surface and SLB. Meanwhile, by placing DIW underneath the SLB, we successively traced potentiometric change by the SLB formation (Fig. 2b), as well as protein bindings above the SLB to the “world’s lowest level of the limit of detection (LOD)” in physiologic conditions of 1× PBS with the SLB (Table 1). The exceptional capabilities of this SLB-FET are attributed to the following elements: (i) defect-free coverage of lipid membrane, (ii) directional alignment of receptors, (iii) prohibition of non-specific binders on the SLB, and most importantly, (iv) ion impermeability of the SLB. Note that the combination of all the ingredients described above resulted in the realization of reliable signal acquisition in solution at physiological ionic strength via the extension of the effective detection range (=Debye length extension) with the vertical shift of stern- and diffusive-layers (Fig. 5). We found that placing the DIW underneath the SLB is the key for shifting the EDL above the outmost surface of the SLB, translating the lipid membrane into a faultless transducer (Fig. 3h). X-ray reflectometry and the corresponding equivalent circuit analysis clearly explained the origin of the obtained electrical responses and their signal transfer mechanism via sub-nanoscale conformational change of lipids during binding events, which were independent from the ionic strengths of the outer buffer solution and even the effective charge states of the bound molecules (Figs. 3h and 4g). In particular, striking FET responses with coterminous amplitudes upon protein bindings with distinct pIs obviously support our findings on the sensing mechanism. Note here that this sensing mechanism by the conformational change in the SLB allows for the detection of electroneutral targets. To the best of our knowledge, our SLB-FET platform presents the first experimental observation of the SLB conformational changes upon molecular binding events with an in-vitro electrical detector, which has been long proposed as a theory but was never experimentally validated. This opens up an opportunity to analyze the physicochemical modulation of lipids at the molecular level, ruling out any possible contribution of the effective charge of target proteins in the signal-transducing mechanism. By resolving the chronic concern of molecular detection in physiologically relevant conditions, our SLB-FET platform and resultant exceptional performance can be extended to identify essential roles of membrane-related pathogenic proteins, and stages/factors of diseases and apoptosis of cells that experience lipid membrane rupture processes. This has wide-ranging implications for conditions such as neurodegenerative disorders, including Alzheimer’s and Parkinson’s diseases^28,29^, virus–cell membrane interaction^30,31^, and the impact of micro-particles on the human respiratory system^32^.

**Table 1:** Comparison of FET sensors for molecule detection.

SLB?	Electron channel	Analyte	LOD	Solvent type & conc.	Refs.
○	Si	Av, S.Av, N.Av	100 pM	1× (100 mM) PBS	Our work
○	CNT	S.Av	2.5 μM	10 mM PBS	^46^
○	CNT	S.Av	5 μM	1 μM PBS	^47^
○	OSC	S.Av	10 nM	10 mM PBS	^48^
○	Graphene	Magainin 2	100 pM	10 mM NaF	^27^
○	Graphene	CTxB	12.5 nM	10 mM HEPES	^49^
×	Si	PSA	75 fg mL^−1^	1 mM PBS, 2 mM KCl	^50^
×	Si	Thrombin	330 pM	Acetate buffer	^51^
×	Si	PSA	150 fM	100 μM PBS, 100 μM KCl	^52^
×	Si	cTnT	1 fg mL^−1^	100 μM PBS	^53^
×	Si	PSA	1 pg mL^−1^	1 μM PBS, 2 μM KCl	^54^

*LOD* limit of detection, *Av* Avidin, *S.Av* Streptavidin, *N.Av* NeutrAvidin, *OSC* organic semiconductor, *PSA* Prostate-specific antigen, *cTnT* Cardiac troponin T.

## Methods

### Fully depleted silicon-on-insulator device fabrication process

Conventional fabrication processes were conducted to construct FET devices, known as Si-nanoribbons^22^ (Supplementary Fig. 1). SIMOX-SOI wafers (100 nm-thick top-Si, Shinetsu) were used to enable top and bottom gating with high precision. In step 1, we selectively etched the top-Si layer using a reactive ion etching (RIE) process to define the active current channel (5 μm long and 30 μm wide) in the FET. A 15-nm-thick oxide layer was formed over the entire top-Si surface using a dry oxidation process (step 2), and confirmed by transmission electron microscope (TEM, Tecnai F20 G2, FEI). For the top gate (TG) electrode, a 200-nm-thick polysilicon (poly-Si) film was deposited, followed by selective etching of the poly-Si and SiO_2_ layers (steps 3 and 4). To minimize the Schottky barrier in metal contacts, arsenic (As) implantation (step 5) followed by annealing (step 6, 850 °C for 30 s) was performed. Then, Ti/TiN/Al/TiN (50/50/250/50 nm) layers were selectively etched using the RIE process, providing soldering pads (steps 7 and 8). Finally, we encapsulated the fabricated devices and mounted them on a printed circuit board with a pin-type interface.

### Faraday shielding box and EG connections

The EG with the reaction chamber was placed in a metallic box for noise shielding. We applied the source and back-gate bias voltages (*V*_S_ and *V*_BG_) with DC voltage sources (GS210, Yokogawa, Japan) with high precision and measured the drain current (*I*_D_) with a digital multimeter (34410A, Agilent, USA). The EG was electrically wired to the TG and terminated with an Ag/AgCl reference electrode (LF-2, Innovative Instruments, Inc., USA) (Supplementary Fig. 2).

### Vesicle preparation and SLB formation

The 1,2-dioleoyl-*sn*-glycero-3-phosphocholine (DOPC: base lipid of the SLB, molecular weight (MW)= 786.1 g mol^−1^) and 1,2-dioleoyl-*sn*-glycero-3-phosphoethanolamine-*N*-(cap biotinyl) (B-PE, MW= 1105.5 g mol^−1^) were purchased from Avanti Polar Lipids Inc. Texas Red 1,2-dihexadecanoyl-*sn*-glycero-3-phosphoethanolamine (TR-DHPE, MW = 1381.85 g mol^−1^) was purchased from Life Technologies, Carlsbad, CA. Avidin variants (avidin, streptavidin, and neutrAvidin, MW= 68, 53, and 60 kDa each.) and various concentrations of PBS solutions were purchased from ThermoFisher Scientific. The SLB membrane consists of 95 mol% DOPC and 5% B-PE (receptor of avidin variants). For vesicle preparation, we used the rapid solvent exchange method to evaporate chloroform and hydrate with buffer solution (DIW or 1× PBS) simultaneously^20^. After that, the prepared vesicle solutions were extruded 20 times through 50-nm-diameter pores (PC membrane, Avanti Polar Lipids Inc., USA) to prepare uniformly sized, small unilamellar vesicles (SUV; 0.1 mg mL^−1^). The 50-nm-diameter SUVs spontaneously rupture on a hydrophilic SiO_2_ surface and form a uniform and defect-free SLB patch on the SiO_2_ surface of the EG^33,34^. For negative controls for biotin–avidin binding, Cholera toxin subunit B proteins (CTxB, Invitrogen, USA, MW= 12 kDa) specifically bound to ganglioside GM1 lipids were used.

### Measurement setup for X-ray reflectivity (XRR)

Synchrotron XRR measurement was carried out using the beamline 5 A, MS-XRS (16.2 keV) in the Pohang Accelerator Laboratory (PAL) with a customized liquid cell. The aluminum liquid cell has Kepton-sealed windows, which are transparent to synchrotron X-ray wavelength, allowing X-ray to be reflected from the surface of the sample while maintaining an aqueous environment inside (Supplementary Fig. 6). The cross-sectional beam size of incident X-ray was 500 × 400 μm^2^, which is large enough to ensure the averaging effect^35^. The SLB (DOPC:B-PE = 95:5) in the DIW was formed via the vesicle fusion method on the 300 nm-thick SiO_2_-coated Si wafer and annealed at 40 °C for 30 min. We injected 800 pM-avidin solution into the SLB and waited for another 30 min to reach equilibrium in the biotin–avidin reaction. After washing to remove protein residues, the wafer was mounted in the liquid cell (Supplementary Fig. 6), which is filled with DIW before the XRR measurement.

### Electron density profile in the SLB obtained from X-ray reflectivity measurement

We used the slab model based on Parratt’s method^36^ (Supplementary Fig. 9) combined with a genetic algorithm to calculate reflectivity. We modeled the SLB with five slabs: two lipid head groups, two lipid tail layers, and a single empty space between each leaflet. For avidin adsorption, four additional slabs were used: three layers of protein and a single layer of DIW between the avidin and the bilayer surfaces. The roughness between each slab was assumed to be 3 Å. Unlike the conventional SLB electron density fitting in which the symmetric electron density profile is assumed, we supposed an asymmetric structure considering that the lipid head group and tail layer in the upper leaflet and bottom leaflet can have different electron densities via avidin adsorption.

### System parameters for all-atom molecular dynamics (AA MD) simulations

We conducted all-atom molecular dynamics simulations in GROMACS 5.1.4^37^ using the CHARMM36 force-field^38^. A leapfrog integrator with a time-step of 2 fs was used for all simulations, restraining all bonds with the LINCS algorithm^39^. The electrostatic interactions of the system were calculated based on the PME with a cutoff of 1.2 nm. Neighbor lists were built using the Verlet cutoff scheme with a cutoff range of 1.2 nm and were updated at every step of the simulations. The temperature was controlled at 298.15 K by a V-rescale thermostat^40^. All systems were maintained at 1 bar using the Berendsen^41^ and Parrinello-Rahman^42^ for the equilibrium and production run, respectively.

### AA MD simulations for SLB on SiO_2_ surface with ionic contrast

We built a binary bilayer made up of DOPC (95%) and B-PE (5%) via the CHARMM-GUI^43^ membrane builder. Ionic contrast between outer buffer and inner buffer can be generated during MD by inserting a SiO_2_ surface. We set up 3 simulation systems with ionic contrast and 1 simulation system without ionic contrast. Overall charge neutrality for all systems was achieved by adding the counter ions. Periodic boundary conditions were applied to *x*, *y*, and *z* directions with an initial cell size of 7.5 × 7.5 × 8 nm^3^. After 100 ns equilibrium steps with pressure coupling, the production simulations were performed for 100 ns to calculate the electron density profile. The electron density profiles of SLB were calculated at the final 10 ns trajectory by the GROMACS analysis tool.

### AA MD for electron redistribution of SLB after avidin binding

The initial structure of the avidin was obtained from the Protein Data Bank (PDB), named 2AVI^44^. For analyzing the electron redistribution after avidin binding, we built the avidin bound binary bilayer system with DOPC/B-PE and the system with DPPC/B-PE. Periodic boundary conditions were applied to *x*, *y*, and *z* directions with an initial cell size of 10 × 10 × 14 nm^3^ and 9 × 9 × 20 nm^3^, respectively. We conducted 400 ns simulations for the equilibration of the structure and 100 ns simulations for analysis. The electron density profiles and dipole potential of avidin bound SLB were calculated by the GROMACS analysis tool. The APL@Voro program^45^ generating the Voronoi diagrams was used for Voronoi analysis and calculation of area per lipids.

## Supplementary information

Supplementary Information

## Data Availability

All relevant data are available from the authors upon reasonable request. Source data are provided with this paper.

## Source data

Source Data
