# Supplementary material for: Ionic contrast across a lipid membrane for Debye length extension: towards an ultimate bioelectronic transducer
Source: Nat Commun. 2021 Jun 18;12:3741. doi: 10.1038/s41467-021-24122-8 (PMC8213817; doi:10.1038/s41467-021-24122-8)
Supplement: Supplementary file 1 — Supplementary Information [file 41467_2021_24122_MOESM1_ESM.pdf]

Supplementary Information for

Ionic contrast across a lipid membrane for Debye length extension : towards an ultimate bioelectronic transducer

Donggeun Lee[1,2,†], Woo Hyuk Jung[3,†], Suho Lee[4], Eui-Sang Yu[1], Taikjin Lee[1], Jae Hun Kim[1], Hyun Seok Song[1], Kwan Hyi Lee[5, 6], Seok Lee[1], Sang-Kook Han[2], Myung Chul Choi[4], Dong June Ahn[3, 6, *], Yong-Sang Ryu[1, 6, *], and Chulki Kim[1,*]

[1]Sensor System Research Center, Korea Institute of Science and Technology, Seoul 02792, Republic of Korea.

[2]Department of Electrical &Electronic Engineering, Yonsei University, Seoul 03722, Republic of Korea.

[3]Department of Chemical and Biological Engineering, Korea University, Seoul 02841, Korea.

[4]Department of Bio and Brain Engineering, Korea Advanced Institute of Science and Technology (KAIST), Daejeon 34141, Republic of Korea.

[5]Center for Biomaterials, Korea Institute of Science and Technology, Seoul 02792, Republic of Korea.

[6]KU-KIST Graduate School of Converging Science and Technology, Korea University, Seoul 02841, Korea.

[†]These authors contributed equally: Donggeun Lee and Woo Hyuk Jung.

*These authors jointly supervised this work: Dong June Ahn, Yong-Sang Ryu, and Chulki kim.

[*]Correspondence to: ahn@korea.ac.kr, ysryu82@kist.re.kr, and chulki.kim@kist.re.kr.

This file contains following information:

Supplementary Notes

1. Device fabrication and measurement setup (Supplementary Figs. 1-3, and Eq. 1)

2. Lipid preparation and FL measurement

3. Buffer solution exchange (Supplementary Fig. 4)

4. Control experiment with identical ionic conditions across the SLB (Supplementary Figs. 5-6)

5. Minimum molecular mass for the full coverage of the SLB (Supplementary Eqs. 2-4)

6. Langmuir–Nernst isotherm model considering nonspecific bindings (Supplementary Eq. 5)

7. Sensing mechanism and analytical models (Supplementary Eqs. 6-11, Fig. 7, and Table 1)

8. X-ray reflectivity measurement for electron density fitting (Supplementary Figs. 8-9)

9. Chemical potential calculation using the electron density (Supplementary Eqs. 12-13, Table 2)

10. Comparison of lipid membranes on a hydrophilic polymer layer vs a SiO_2 layer (Supplementary Fig. 10)

11. Molecular dynamics (MD) simulation for investigating the conformational change (Supplementary Fig. 11-12)

Supplementary References

Supplementary Note 1. Device fabrication and measurement setup

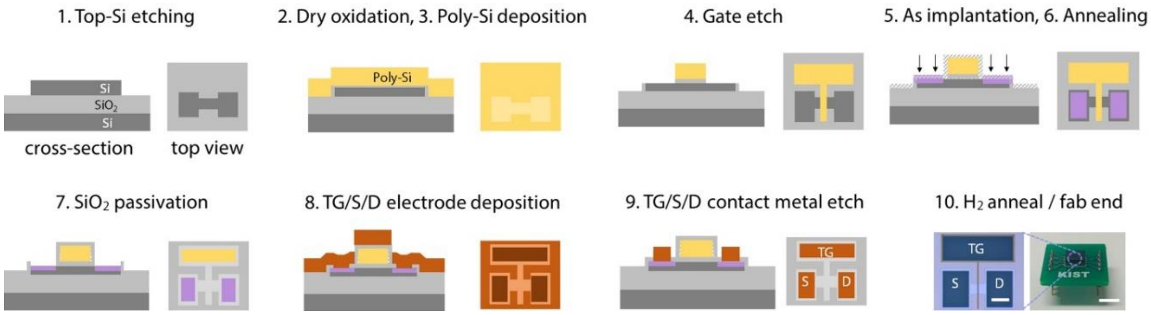

Supplementary Fig. 1. Schematic representation of fabrication of FETs for molecular detection

Extended gate (EG) fabrication: The top gate electrode of the FET is wired to an indium tin oxide (ITO, thickness: 300 nm and resistivity: 5 Ω/sq) electrode. A 80-nm-thick SiO_2 layer was deposited on the ITO for bonding the reaction chamber to the ITO electrode. The reaction chamber was made of polydimethylsiloxane (PDMS, Sylgard 184 silicon elastomer kit, Dow Corning). The PDMS mixture (base:curing agent = 10:1) was poured into a Petridish and baked for 12 h at 80℃. After curing, an 8-mm diameter and a 5-mm height hole was formed in the PDMS mold using a biopsy punch. Note that the SiO_2 layer plays a bifunctional role here: (1) a surface for forming the supported lipid bilayer (SLB) membrane and (2) serving as a hydrophilic polar silanol (Si-OH) group cross-linked surface for the PDMS chamber via covalent bonding after an oxygen plasma treatment[1].

Importance of disposable EG: The EG reaction chamber is disposable. This measurement configuration for the EG and the remotely packaged FET allows for reliable data acquisition, eliminating any possible contamination or damage to the active current channel upon direct exposure to target analytes in ionic solution. Moreover, direct comparison between measurements is possible because an identical FET transduces signals from the disposable EG. This is important during quantification of analyte concentration.

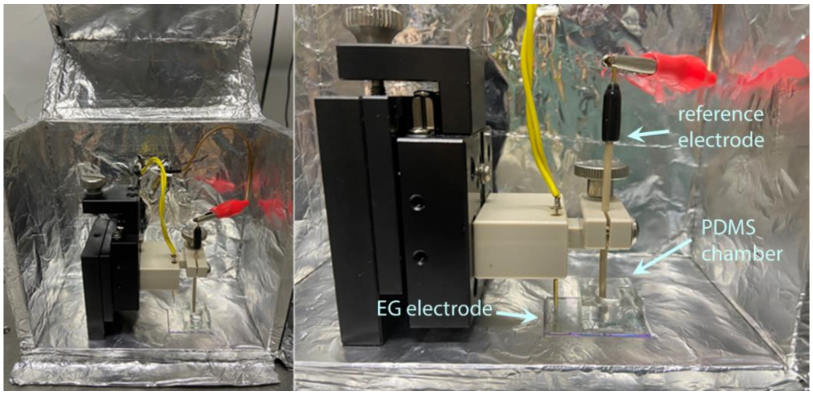

Supplementary Fig. 2. Photograph images of the EG with the reaction chamber placed in a Faraday shielding box

Back gate bias voltage conditions: In our measurement configuration, the potential variation ($\Delta\psi$) of the sensing probe (EG) is capacitively coupled to top gate voltage (V_{TG}), modulating drain current (I_D) in the FET. The proportionality between the variations of V_{TG} and I_D is defined by transconductance (g_m),

$$g_{\mathrm{m,max}} = g_{\mathrm{m}}|_{V_{\mathrm{TG}}=V_{\mathrm{th}}} = \frac{\mathrm{d}I_{\mathrm{D}}}{\mathrm{d}V_{\mathrm{TG}}} \quad (1)$$

where $\mathrm{d}I_D$ and $\mathrm{d}V_{TG}$ are the small variation of the drain current and the top gate voltage. As seen in the Supplementary Eq. 1, the g_m represents the amplification of the output response in the transducing process from the molecular bindings. Namely, the $\mathrm{d}V_{TG}$ caused by the molecular binding is transduced into the obtained current variation $\mathrm{d}I_D$. The g_m is typically maximized at around the threshold voltage V_{th}. However, this bias voltage condition to maximize the g_m is affected by the ionic strength of the analyte solution.

In our dual gate configuration, we can address this issue systematically by tuning back gate bias voltage V_{BG}. As shown in Supplementary Fig. 3, the threshold voltage (=$g_{m,max}$) can be shifted by the V_{BG}. Thus, we characterized the FET in the measurement condition to determine the back gate bias voltage for the maximized g_m. With this optimizing process, the applied g_m is ensured to be maximized throughout the measurements. For instance, the g_m was maximized with the V_{BG} of 13.5 V in the DIW environment.

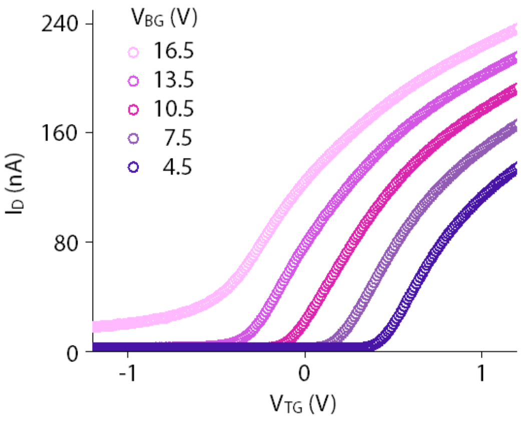

Supplementary Fig. 3. I_D-V_{TG} curves at various back gate voltages V_{BG}. ($V_S = 1$ V)

<u>Supplementary Note 2. Lipid preparation and fluorescence (FL) measurement</u>

FL imaging and mobility test: For the FL imaging, we monitored the SLB using an exposure time of 100 ms and gain 5.8× for every FL micrograph in the SLB coverage experiments. The FL and bright-field (BF) images of particulate materials were monitored using a FL microscopy system (LV100ND, Nikon) and then analyzed using image processing software (ImageJ, National Institutes of Health, USA). For the FRAP test, a 20-μm-circle region of the SLB patch was bleached for 1 min, and time-lapse FL intensities were measured using confocal microscopes (C2 C-ER, Nikon, Japan).

Supplementary Note 3. Buffer solution exchange

After the SLB (DOPC:B-PE = 95:5) preparation in DIW, half of the solution in the EG reaction chamber (50 μL out of 100 μL) was exchanged with 50 μL of 1× PBS. This process was repeated until the V_{TG} value was saturated (Supplementary Fig. 4). Note that the signal reached a steady state after 10 repeated exchanges of the outer buffer (OB), which is consistent with the expected concentrations; 0.992× PBS at the 7th buffer solution exchange and 0.999× PBS at the 10th buffer solution exchange (Supplementary Fig. 4).

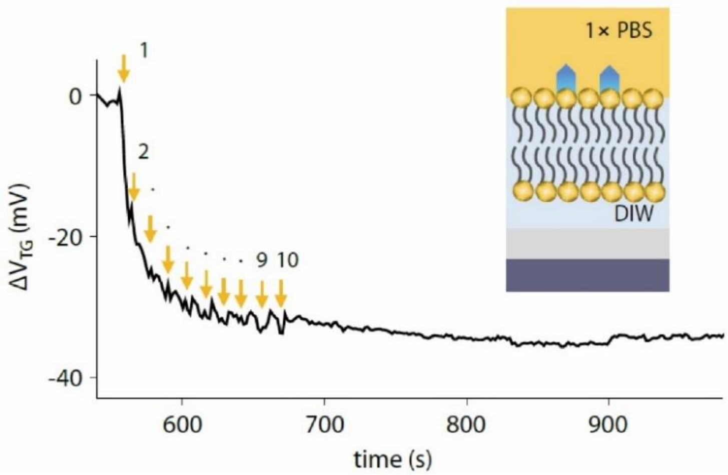

Supplementary Fig. 4. Potential variation of the top gate electrode during the OB exchange process with the ionic contrast across the SLB

Supplementary Note 4. Control experiment with identical ionic conditions across the SLB

To validate the role of the DIW layer between the SiO_2 surface and the SLB, a negative control experiment was carried out using symmetrical ionic conditions across the SLB. Namely, the SUV was prepared in 1× PBS (0.1 mg/mL) and exposed to the hydrophilic SiO_2 surface of the EG. After SLB formation (marker 2 in Supplementary Fig. 5), we waited for at least 5 min for signal stabilization. No signal change was observed after the 800 pM-avidin injection (marker 3 in Supplementary Fig. 5), in contrast to the recognizable ΔV_{TG} changes under 800 pM-avidin bindings under asymmetric ionic conditions across the SLB (main manuscript Figs. 3a and h). This is attribute to the ultra-thin Debye length (approximately 0.74 nm in 1× PBS), which is thinner than IB layer (1–2 nm) between SLB and ITO surface.

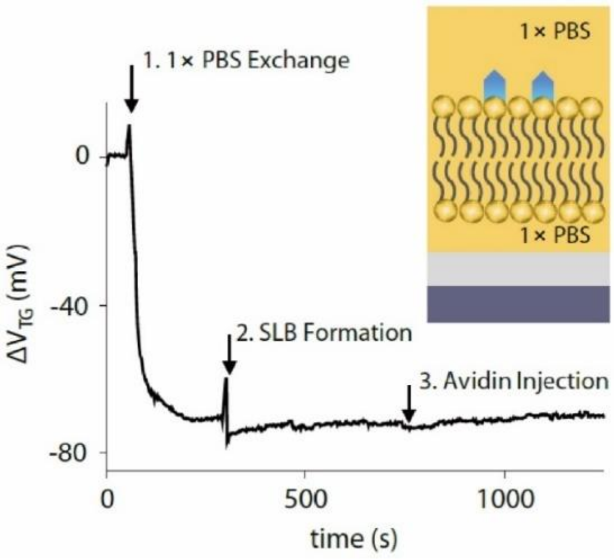

Supplementary Fig. 5. Real-time ΔV_{TG} measurement of the response to biotin–avidin reactions under identical ionic conditions across the SLB

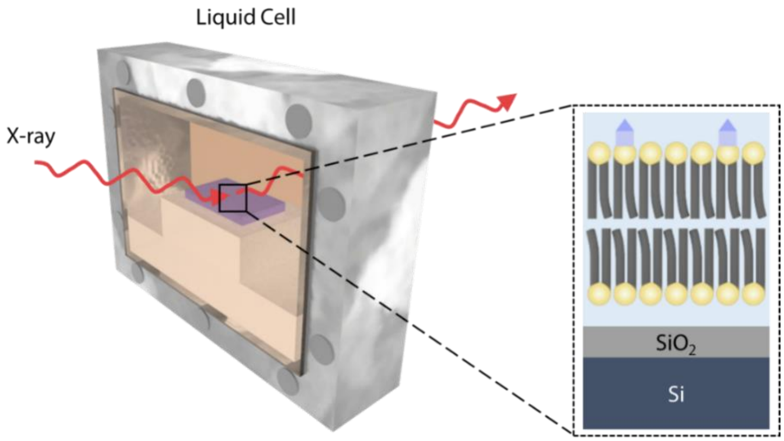

Supplementary Fig. 6. Representation of the liquid cell for X-ray reflectivity measurement and the SLB on a SiO_2 wafer placed in the cell

Supplementary Note 5. Minimum molecular mass for the full coverage of the SLB

We estimated the required number of lipid molecules to cover the entire surface of the SiO_2 and the inside wall of the reaction chamber A_{EG},

$$A_{\mathrm{EG}} = \pi r^2 + 2\pi r h = 125.27\ (\mathrm{mm}^2) \tag{2}$$

where r and h are the diameter and height of the EG chamber, respectively. Considering the head area of DOPC molecules[2] (A_{SLB} = 72.4 Å ^{2}), the required number of DOPC molecules was calculated to be

$$\frac{A_{\mathrm{EG}}}{A_{SLB}/2} = \frac{125.27\ (mm^2)}{36.2\ (\text{Å})} = 0.57 \times 10^{-9}\ (\mathrm{mol}). \tag{3}$$

With an SLB composition of DOPC/B-PE = 95/5, the averaged mass of the SLB is

$$\Bigg(\underbrace{786.1 \times 0.95}_{DOPC} + \underbrace{1105.5 \times 0.05}_{B-PE}\Bigg)(g\ mol^{-1}) \times (0.57 \times 10^{-9})(mol) \approx 0.46\ \mu g. \tag{4}$$

This means that 0.46 μg of lipids were required for full coverage of the EG including the PDMS wall and the SiO_2 surface. Considering that the mass of the lipid in solution was 0.1 μg for every drop, the observed saturation after five lipid injections (markers 2 to 6 in main manuscript Fig. 2b) seems reasonable amount for the full coverage of the EG surface. Additional casting of lipid molecules (marker 7 in the main manuscript Fig. 2b) confirms that 5 times of lipid introduction was sufficient for the complete passivation of the EG surface.

Supplementary Note 6. Langmuir–Nernst isotherm model considering nonspecific bindings

The SLB allows covering the EG surface, and thereby prohibit the nonspecific binding of analytes to the EG surface. To investigate and quantify the role of SLB as the 'suppressor' for the nonspecific binding, we conducted an experiment with the EG functionalized by sulfo-NHS-biotin and compared this with the isotherm analysis of the SLB-FET (main manuscript Fig. 3d). The Langmuir–Nernst isotherm model is described as follows:

$$\Delta V_{\mathrm{TG}}([A]) = \frac{q_A}{C_{TG}}[B]_{\max}\frac{[A]}{[A]+K_{eq}} + k_N[A] \tag{5}$$

where K_{eq}, and k_N stand for equilibrium constant and nonspecific binding constant, respectively[3].

The strong suppression of nonspecific binding in our SLB-FET is described in the main manuscript in Fig. 3d. The measured result (circles in orange in main manuscript Fig. 3d) from our SLB-FET is highly consistent with the conventional Langmuir isotherm model (solid orange line in main manuscript Fig. 3d, $k_N[A] = 0$). Biotin–avidin binding signals measured with the sulfo-NHS-biotin functionalized EG (circles in grey in main manuscript Fig. 3d) exhibited strong agreement with the Langmuir–Nernst isotherm (solid grey line in main manuscript Fig. 3d). From the fitting, k_N and K_{eq} were calculated to be 3.685 ± 0.754 mV/μM·m and 54.2 fM, respectively, which was within the reasonable range[4]. Note that our SLB-FET platform allowed for negligence of the second term, whereas protein bindings without SLB should take into account the possibility of nonspecific binding.

Supplementary Note 7. Sensing mechanism and analytical models

Molecular detection using FETs is based on conductivity changes in response to variance in the gate potential. In our case, using an n-type channel for FET and applying a positive gate voltage leads to accumulation of carriers. When analytes bind to receptors on the SLB, the charge density variation in the SiO_2 layer changes the top gate voltage, and eventually the conductance in the FET. The time-dependent top gate voltage induced by adsorbed molecules is described by Supplementary Eq. 6,

$$\frac{\mathrm{d}\Delta V_{\mathrm{TG}}(t)}{dt} = \frac{q_A}{C_{\mathrm{TG}}}[AB] \tag{6}$$

where $[AB]$ is the density of an adsorbed analyte.

In general, the binding kinetics of the receptor when capturing an analyte in solution can be described as follows:

$$[A] \overset{k_M}{\leftrightarrow} [A]_S + [B] \xleftrightarrow{k_1,\ k_{-1}} [AB] \tag{7}$$

where k_M is a diffusion-limiting rate constant, k_1 and k_{-1} are association and dissociation rate constants, respectively, and $[A]_s$ is the surface concentration of an analyte[5]. We assumed the fast mixing model ($[A]_S = [A]$) to describe our SLB-FET system, because the k_1 in the biotin–avidin reaction (10^5 to 10^7 $M^{-1}s^{-1}$) is two or more orders of magnitude smaller than the k_M (~10^9 $M^{-1}s^{-1}$) for diffusion limited reactions[6]. In other words, we suppose that the analyte concentration at the surface remains almost the same as in the bulk in the following analysis. With this, the reaction can be described in a simpler fashion by the first order Langmuir equation (Supplementary Eq. 8a).

$$\frac{\mathrm{d}[AB]}{\mathrm{d}t} = k_1[A]([B]_{\max} - [AB]) + k_{-1}[AB] \tag{8a}$$

Then, the density of the adsorbed analyte is explicitly expressed by Supplementary Eq. 8b.

$$[AB]_{\mathrm{t}} = \frac{\mathrm{k}_1[B]_{\mathrm{max}}[A]}{k_1[A] + k_{-1}}\left(1 - e^{-(k_1[A]+k_{-1})t}\right) = [B]_{\mathrm{max}}\frac{[A]}{[A] + K_{\mathrm{eq}}}\left(1 - e^{-(k_1[A]+k_{-1})t}\right) \quad (8b)$$

By combining Eqs. 6 and 8b, the time-dependent voltage variation is described by Supplementary Eq. 9,

$$\Delta V_{\mathrm{TG\_adsorption}}(t) = \frac{q_{\mathrm{A}}}{C_{\mathrm{TG}}}[B]_{\mathrm{max}}\left(1 - e^{-(k_1[A]+k_{-1})t}\right) \quad (9)$$

In our measurements, the target analyte in solution is dropped in the reaction chamber; therefore, this inevitably leads to a sudden increase in the ionic concentration in the outer buffer. Thus, the top gate potential should reflect relaxation behavior via thermal diffusion. We model its transient state towards equilibrium by a resistor–capacitor (RC) circuit model as in Supplementary Eq. 10.

$$\Delta V_{\mathrm{TG\_RC}}(t) = \begin{cases} V_p(1 - e^{-\frac{t}{\tau_1}}) & t < T \\ V_p e^{-\frac{t}{\tau_2}} & t \geq T \end{cases} \quad (10)$$

where the peak voltage V_p is the difference between $q_A[B]_{\mathrm{max}}/C_{\mathrm{TG}}$ and the maximum value of $\Delta V_{\mathrm{TG}}(t)$, τ_1 and τ_2 are RC time constants, and T is an effective duration for the sudden increase in the ionic concentration in the outer buffer.

Finally, the measured signal should be the superposition of these responses described above (Eqs. 9 and 10).

$$\Delta V_{\mathrm{TG}}(t) = \begin{cases} \frac{q_A}{C_{\mathrm{TG}}}[B]_{\mathrm{max}}\left(1 - e^{-(k_1[A]+k_{-1})t}\right) + V_p(1 - e^{-\frac{t}{\tau_1}}) & t < T \\ \frac{q_A}{C_{\mathrm{TG}}}[B]_{\mathrm{max}}\left(1 - e^{-(k_1[A]+k_{-1})t}\right) + V_p e^{-\frac{t}{\tau_2}} & t \geq T \end{cases} \quad (11)$$

Supplementary Fig. 7 shows the response curve for 100 pM-avidin binding over the SLB (DOPC:B-PE = 95:5), fitted to our suggested theory model. From these fittings, important reaction parameters are obtained. Those parameters are summarized in Supplementary Table 1.

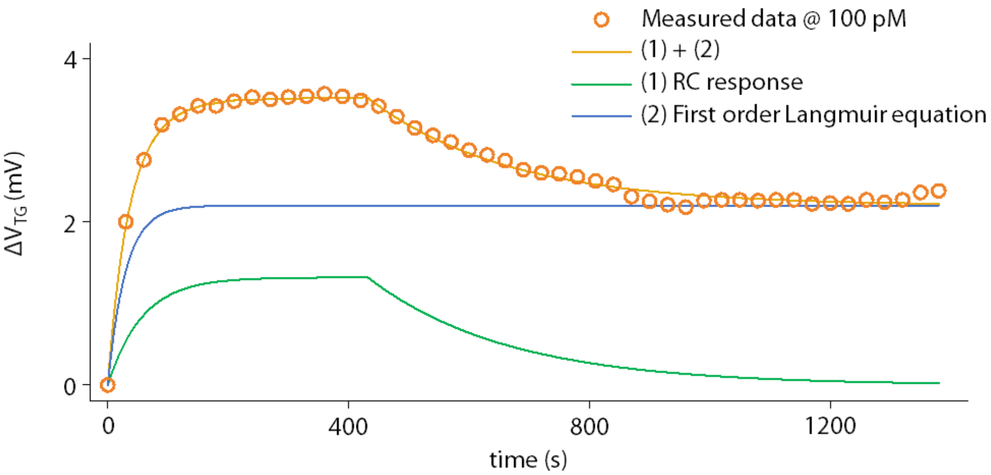

Supplementary Fig. 7. Real-time top gate voltage variation in response to 100 *pM*-avidin fitted by an RC circuit model and the first order Langmuir equation.

Supplementary Table 1. Fitting parameters in Supplementary Eq. 10

	V_p (mV)	τ_1 (sec)	V_{eq} (V)	k_1 ($\times 10^7 M^{-1}s^{-1}$)	k_{-1} ($\times 10^{-2} s^{-1}$)	τ_2 (sec)	T (sec)
100 pM	1.3200	29.682	0.0022	1.6835	1.0469	231.790	432.984
1 nM	0.7802	12.971	0.0053	1.6642	1.0350	106.525	219.648
5 nM	-	-	0.0079	1.6606	1.0311	-	-
10 nM	-	-	0.0088	1.6227	1.0010	-	-
100 nM	-	-	0.0089	1.7163	1.0410	-	-

Supplementary Note 8. X-ray reflectivity measurement for electron density fitting

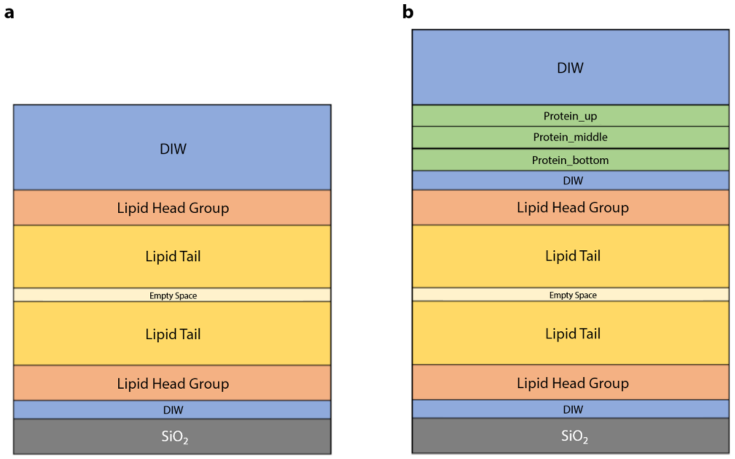

Supplementary Fig. 8. Structure for a slab model. a, the SLB and **b**, the SLB with a layer of avidin molecules

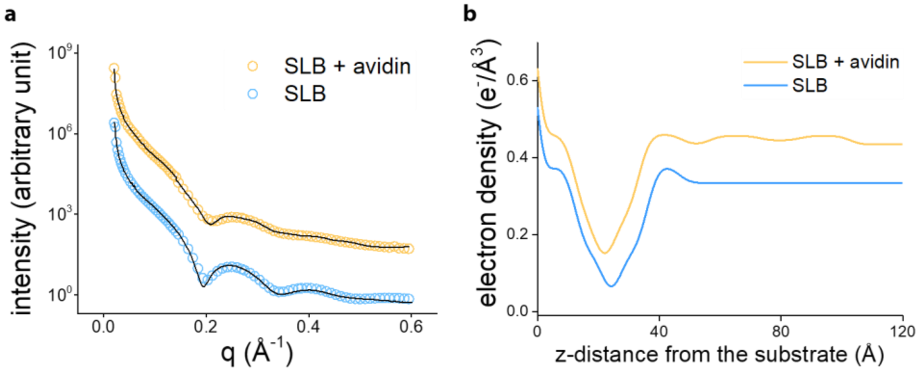

Supplementary Fig. 9. X-ray intensities and electron density profiles of SLB (blue) and SLB with avidin (yellow). a, X-ray reflectivity intensities and their fittings. **b**, Electron density curves along the z-direction.

As shown in Supplementary Fig. 9a, both measurements of ‘SLB’ and ‘SLB with avidin’ were well fitted by the model described above. Slight deviation at high q regime (>0.5 Å^{-1}) is due to the low peak intensity. The electron density plots in Supplementary Fig. 9b clearly exhibit the typical electron density curve of SLB, showing consistency with the literature in terms of the size of the conjugated avidin[2] (~50 Å).

Supplementary Note 9. Chemical potential calculation using the electron density

Poisson's equation (Supplementary Eq. 12) was applied to calculate electric potential variation due to the redistribution of the charge density in the lipid membrane[7, 8, 9].

$$\psi(\mathrm{z}) - \psi(0) = -\frac{1}{\varepsilon_0}\int_0^z dz' \int_0^{z'} \rho(z'')dz'' \tag{12}$$

The chemical potential at the probing surface was calculated by integrating the charge density ($\Delta\rho = \rho_{\mathrm{SLB}} - \rho_{\mathrm{SLB+avidin}}$), and multiplied by the area of SLB film ($\pi \times R^2 \approx 5.027 \times 10^{-5}$ m^2) assuming in-plane homogeneity.

$$\Delta\psi(\mathrm{z}) - \Delta\psi(0)$$

$$= -\frac{1}{\varepsilon_0}\int_0^z \int_0^{z'} \Delta\rho(z'')dz''\,dz'$$

$$= -\frac{\mathrm{e}^- \times \pi r^2}{\varepsilon_0}\int_0^z \int_0^{z'} \Delta\rho(z'')dz''\,dz'$$

$$= -\frac{1.602 \times 10^{-19} \times 5.027 \times 10^{-5}}{8.854 \times 10^{-12}}\int_0^z \Delta E(z') - \Delta E(0)dz' = 0.697\ (\mathrm{V}) \tag{13}$$

As seen in the main manuscript Fig. 4d, the obtained signal mainly originates from the charge density change in proximity of the probing surface because the chemical potential is inversely proportional to the distance squared.

Supplementary Table 2. Electric parameters of FET-SLB circuit components

	C_{EG}	C_{TG}	C_{BG}	C_{SLB}
Dielectric constant (ε_r)	3.9	3.9	3.9	1.9
Area (m^2)	5.03E-05	1.50E-10	1.50E-10	5.03E-05
thickness (m)	8.00E-08	1.50E-08	7.50E-07	5.00E-09
Capacitance (F)	2.17E-08	3.45E-13	6.91E-15	4.36E-06
Voltage (V)	2.18E-07	1.37E-02	6.83E-01	1.08E-09

Supplementary Note 10. Comparison of lipid membranes on a hydrophilic polymer layer vs a SiO_2 layer

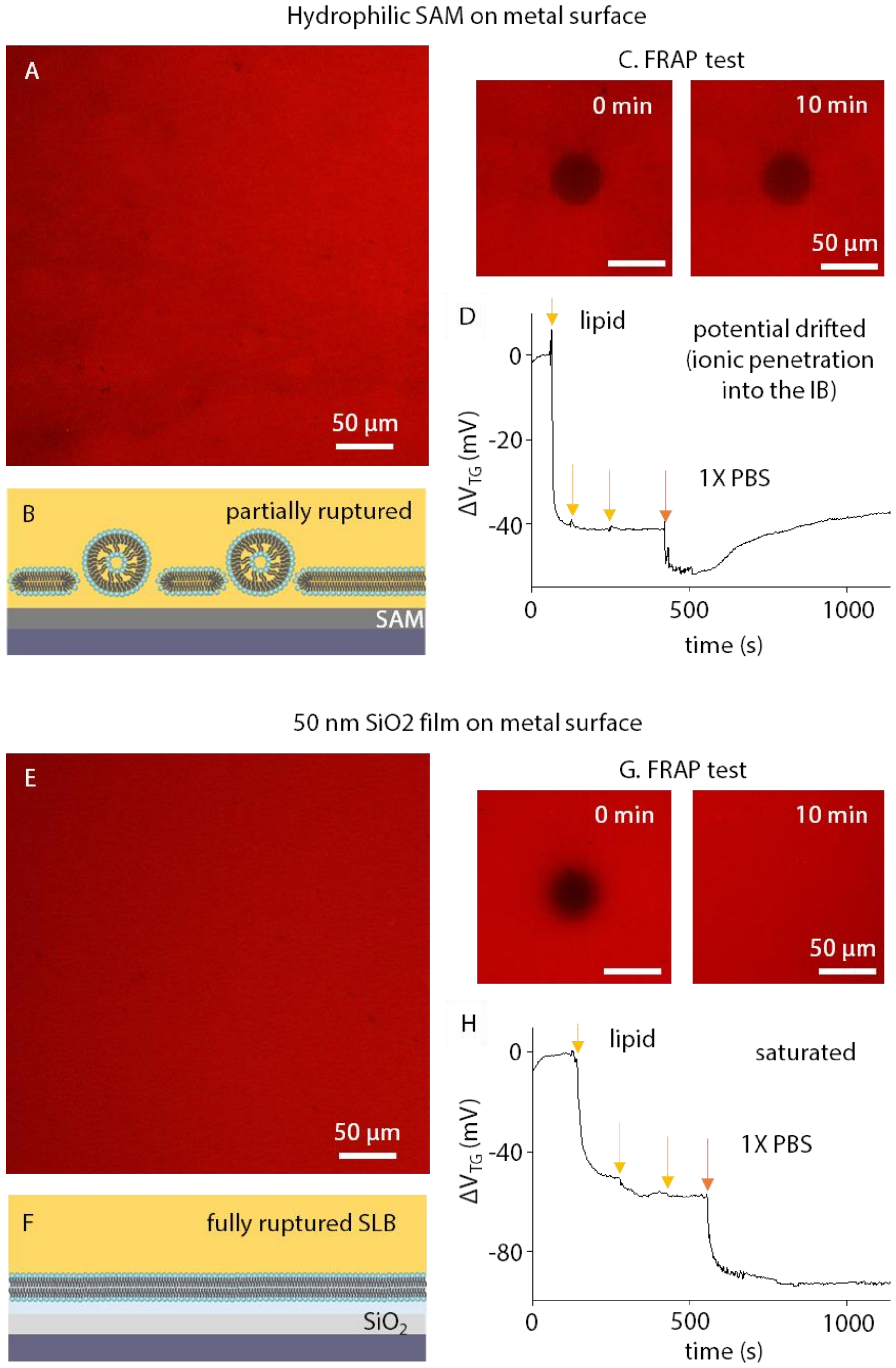

Supplementary Fig. 10. Direct comparison of lipid membrane over the hydrophilic polymer vs the SiO_2 layer. a, Non-uniform FL distribution of supported lipid membrane on the hydrophilic

polymer (2-Mercaptoethanol) on the EG. **b**, corresponding schematic illustration. **c**, Partial immobility of lipid compositions during a FRAP test. Albeit become brighter, the circular FL bleached area does completely recovered in 10 min. **d**, Time-dependent ΔV_{TG} after the outer buffer exchange (DIW → 1× PBS) showing the leakage flow of ions through the pin-hole of the lipid membrane. **e**, **f**, Uniform SLB formation over the 50-nm-SiO_2 layer (e) with a schematic illustration (f). **g,** FRAP test showing later fluidity of the composing lipids. Note that the FL bleached (black) region disappeared within 10 min. **h**, Time-lapse ΔV_{TG} variation after the OB exchange (DIW → 1× PBS). The ΔV_{TG} was saturated after the ionic contrast between IB (DIW) and OB (1× PBS) was obtained.

This is the major reason why we prepared the SLB on the SiO_2 film. Although mass production of biosensors using polymeric SAM seems far-fetched, our suggested SLB-FET with the SiO_2 film on the EG guarantees the robust formation of the SLB in terms of coverage (Supplementary Fig. 10e) and lipid mobility (Supplementary Fig. 10g). Most importantly, stable OB exchange manifests the importance of the defect-free SLB. This allows reliable molecular detection with high reproducibility in real time (Supplementary Fig. 10h).

Supplementary Note 11. Molecular dynamics (MD) simulation for investigating the conformational change

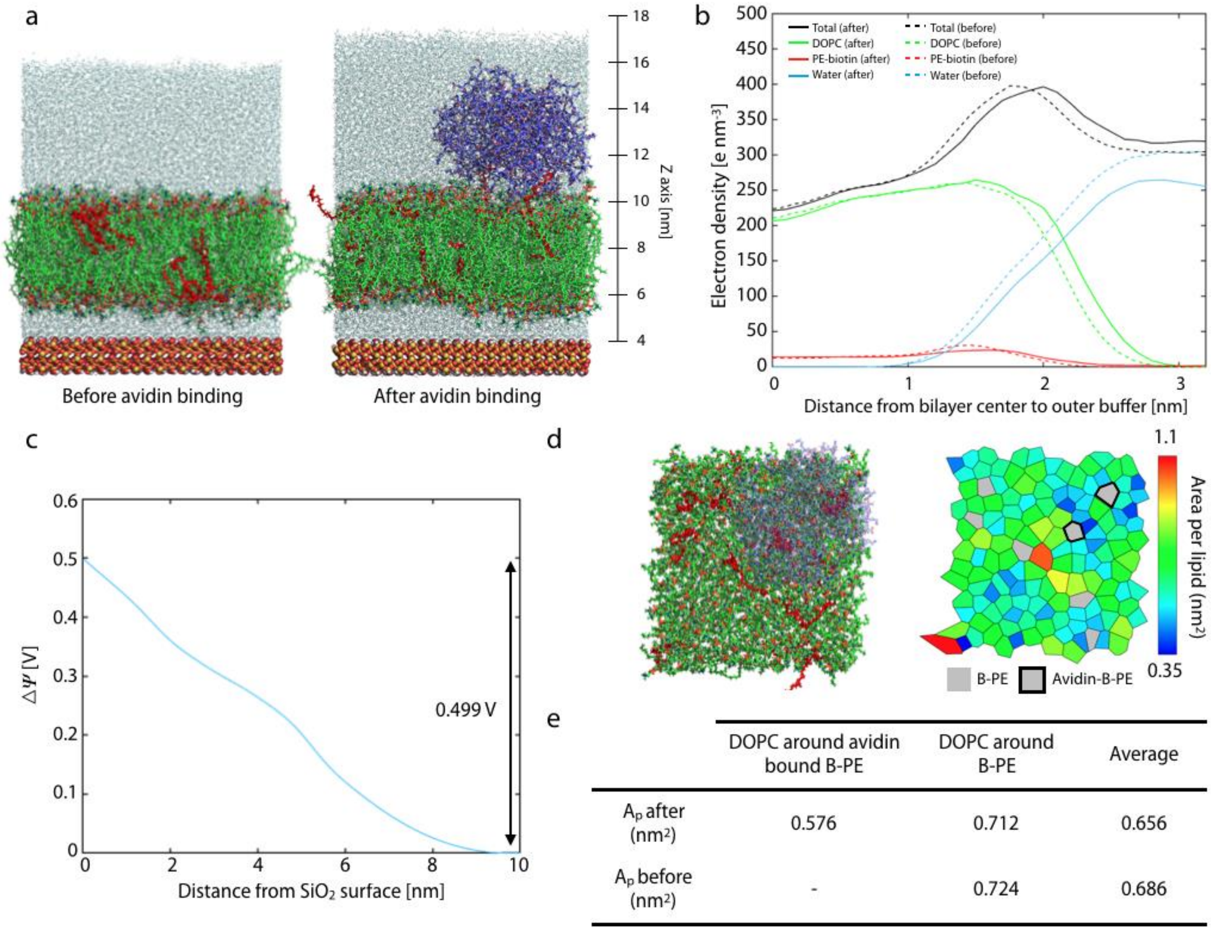

	DOPC around avidin bound B-PE	DOPC around B-PE	Average
A_p after (nm^2)	0.576	0.712	0.656
A_p before (nm^2)	-	0.724	0.686

Supplementary Fig. 11. MD simulation on electron density and conformational change in the SLB membrane (DOPC:B-PE=95:5) upon biotin-avidin binding. a. Final configurations of the SLB before (left) and after (right) avidin binding. The DOPC lipids, B-PE lipids, avidin, and water molecules are represented by the green, red, purple, and cyan colours, respectively. The SiO_2 surface are represented by red (O atoms) and yellow (*Si* atoms) spheres. **b**. Electron density profiles of the upper leaflet in the membrane before and after biotin-avidin bindings. **c**. Potential difference ($\Delta\psi$) upon biotin-avidin binding calculated by Poisson's equation. **d**. Top view of avidin-bound membrane and corresponding area per lipid calculated by Voronoi analysis. **e**. Chart for calculated areas per lipid for constituent lipids.

Supplementary Fig. 11 shows the obtained simulation results for the final configurations of binary lipid membrane (DOPC:B-PE=95:5) upon avidin binding events under a symmetrical ionic condition across the lipid bilayer (DIW for both of IB and OB). We then calculated the electron density profile of lipids to investigate whether the avidin-biotin binding event can modulate the profiles of the lipid membrane. The electron density profile achieved from the centre of the lipid membrane shows that the maximum peak of electron density at the upper leaflet of the lipid bilayer shifts toward a higher value of z after avidin binding, indicating slight thickening of the lipid bilayer (Supplementary Fig. 11b). For quantitative comparison with experimental results, the potential difference, $\Delta\psi$, via the avidin binding was calculated using Poisson's equation (Supplementary Fig. 11c). As a result, the avidin bound membrane induced chemical potential change of $\Delta\psi$ = 499 mV at the surface of the SiO_2 layer. This agrees very well with the experimental results shown in main Fig. 4d. We further measured the structural characteristic of the lipid bilayer, the area per lipid, A_p, to quantify the conformational change of the membrane upon avidin binding. Top view of the avidin bound DOPC/B-PE membrane and corresponding Voronoi cells represented for DOPC and B-PE are shown in Supplementary Fig. 11d. This shows in-plane (x-y plane) compression of lipid membrane when two biotins bound for two binding sites of an avidin (indicated as 'Avidin-B-PE', Supplementary Fig. 11d). The color map analyzed in the Voronoi cell (Supplementary Fig. 11d) and corresponding data in area per lipid (Supplementary Fig. 11e) clearly indicate that avidin bound B-PEs become the seed spots causing the membrane packing effect (a decreased area per lipid). Successive decrease of the area per lipid around the avidin bound B-PE accompanies the thickening effect over the lipid membrane, which results in a potential decrease of the SiO_2 surface. Considering that the packing density is one of the essential criteria in defining the modulation of the lipid membrane during the avidin binding, the MD

simulation results with DOPC/B-PE lipid mixtures shows great correspondence with the electron density profiles obtained from XRR.

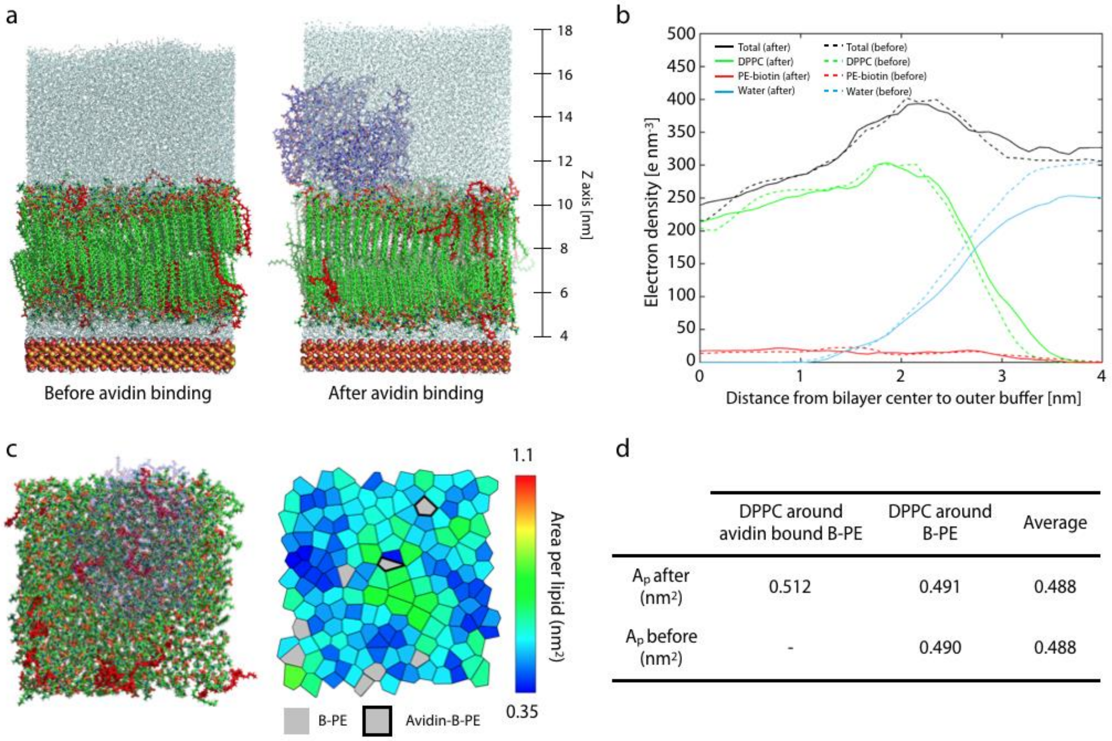

	DPPC around avidin bound B-PE	DPPC around B-PE	Average
A_p after (nm^2)	0.512	0.491	0.488
A_p before (nm^2)	-	0.490	0.488

Supplementary Fig. 12. MD simulation on electron density and conformational change in the SLB (DPPC:B-PE=95:5) upon biotin-avidin binding. a. Final configurations of the SLB before (left) and after (right) avidin binding. The DPPC lipids, B-PE lipids, avidin, and water molecules are represented by the green, red, purple, and cyan colours, respectively. The SiO_2 surface are represented by red (O atoms) and yellow (Si atoms) spheres. **b**. Electron density profiles of the upper leaflet in the membrane before and after biotin-avidin bindings. **c**. Top view of the avidin-bound membrane and corresponding area per lipid calculated by Voronoi analysis. **d**. Chart for calculated areas per lipid for constituent lipids.

Supplementary Fig. 12a shows the final configurations of the DPPC lipid mixed with B-PE (DPPC:B-PE=95:5) before and after the avidin binding. In contrast to the membrane with the mobile DOPC lipid, the stiffer and more crystalized DPPC with B-PE shows a negligible effect on the modulation of the electron density profile (Supplementary Fig. 12b). Top view of the avidin bound DPPC/B-PE membrane, and corresponding Voronoi cells represented the area per lipid, A_p, for DPPC and B-PE are shown in Supplementary Fig. 12c. The area per lipid calculated from the Voronoi cells represents that negligible conformational change of the lipid membrane was observed in the binary DPPC/B-PE membrane after avidin binding (Supplementary Fig. 12d). In summary, insignificant physicochemical modulation was observed in the DPPC/B-PE under avidin binding.

Supplementary References

1. Y. S. Ryu *et al.*, Kinetics of lipid raft formation at lipid monolayer-bilayer junction probed by surface plasmon resonance. *Biosens. Bioelectron.* **142**, 111568 (2019).
2. N. Kucerka, S. Tristram-Nagle, J. F. Nagle, Structure of fully hydrated fluid phase lipid bilayers with monounsaturated chains. *J. Membrane Biol.* **208**, 193-202 (2005).
3. L. Maletinska et al., Human glioblastoma cell lines: Levels of low-density lipoprotein receptor and low-density lipoprotein receptor-related protein. *Cancer Res.* **60**, 2300-2303 (2000).
4. R. A. Kohanski, M. D. Lane, Monovalent avidin affinity columns. *Methods Enzymol.* **184**, 194-200 (1990).
5. X. Duan et al., Quantification of the affinities and kinetics of protein interactions using silicon nanowire biosensors. *Nat. Nanotechnol.* **7**, 401-407 (2012).
6. R. F. Delgadillo *et al.*, Detailed characterization of the solution kinetics and thermodynamics of biotin, biocytin and HABA binding to avidin and streptavidin. *PLoS One* **14**, e0204194–e0204194 (2019).
7. I. D. Mayergoyz, Solution of the nonlinear Poisson equation of semiconductor-device theory. *J. Appl. Phys.* **59**, 195-199 (1986).
8. H. J. Shen *et al.*, Molecular dynamics simulations of ether- and ester-linked phospholipid Bilayers: A comparative study of water models. *J. Phys. Chem. B* **122**, 9399-9408 (2018).
9. K. E. Forsten, R. E. Kozack, D. A. Lauffenburger, S. Subramaniam, Numerical solution of the nonlinear Poisson-Boltzmann equation for a membrane-electrolyte system. *J. Phys. Chem.* **98**, 5580-5586 (1994).
